# Sulforaphane Delays Fibroblast Senescence by Curbing Cellular Glucose Uptake, Increased Glycolysis, and Oxidative Damage

**DOI:** 10.1155/2018/5642148

**Published:** 2018-11-22

**Authors:** Florence Hariton, Mingzhan Xue, Naila Rabbani, Mark Fowler, Paul J. Thornalley

**Affiliations:** ^1^Clinical Sciences Research Laboratories, Warwick Medical School, University of Warwick, University Hospital, Coventry CV2 2DX, UK; ^2^Unilever Research & Development Colworth, Sharnbrook, Bedford MK44 1LQ, UK; ^3^Diabetes Research Center, Qatar Biomedical Research Institute, Hamad Bin Khalifa University, Qatar Foundation, P.O. Box 5825, Doha, Qatar

## Abstract

Increased cell senescence contributes to the pathogenesis of aging and aging-related disease. Senescence of human fibroblasts *in vitro* may be delayed by culture in low glucose concentration. There is also accumulating evidence of senescence delay by exposure to dietary bioactive compounds that activate transcription factor Nrf2. The mechanism of cell senescence delay and connection between these responses is unknown. We describe herein that the cruciferous vegetable-derived metabolite, sulforaphane (SFN), activates Nrf2 and delays senescence of human MRC-5 and BJ fibroblasts *in vitro*. Cell senescence is associated with a progressive and marked increased rate of glucose metabolism through glycolysis. This increases mitochondrial dysfunction and overwhelms defences against reactive metabolites, leading to increasing proteomic and genomic oxidative damage. Increased glucose entry into glycolysis in fibroblast senescence is mainly mediated by increased hexokinase-2. SFN delayed senescence by decreasing glucose metabolism on the approach to senescence, exhibiting a caloric restriction mimetic-like activity and thereby decreased oxidative damage to cell protein and DNA. This was associated with increased expression of thioredoxin-interacting protein, curbing entry of glucose into cells; decreased hexokinase-2, curbing entry of glucose into cellular metabolism; decreased 6-phosphofructo-2-kinase, downregulating formation of allosteric enhancer of glycolysis fructose-2,6-bisphosphate; and increased glucose-6-phosphate dehydrogenase, downregulating carbohydrate response element- (ChRE-) mediated transcriptional enhancement of glycolysis by Mondo/Mlx. SFN also enhanced clearance of proteins cross-linked by transglutaminase which otherwise increased in senescence. This suggests that screening of compounds to counter senescence-associated glycolytic overload may be an effective strategy to identify compounds with antisenescence activity and health beneficial effects of SFN in longevity may involve delay of senescence through glucose and glycolytic restriction response.

## 1. Introduction

Human diploid fibroblasts exhibit a progressive decreased replication rate in culture, eventually reaching a maximum cumulative number of cell replications called the limiting population doubling level or Hayflick limit [1]. This process is called replicative senescence (RS). It reflects the dysfunction of mitotic control of dividing cells and reflects the aging process, with also metabolic relevance to the aging of postmitotic cells. Phenotypic characteristics of the approach to RS are exit from the cell cycle, unresponsiveness to growth factors, shortening of telomeres, increase in cell size and heterogeneity, expression of *β*-galactosidase (*β*-gal), senescence-associated secreted phenotype (SASP), and others [2]. Senescence may be activated independent of shortening of telomers where an abnormal response to DNA replication stress or telomer damage may be involved [3, 4]. The relevance of the RS model to human ageing is claimed through the observation of increased *β*-gal activity in the skin of elderly people [5] and association of cell senescence in human disease [6]. Delay of senescence weakens engagement of cells in senescence pathways or blocks mediators of RS.

Senescence of human fibroblasts *in vitro* is delayed by culture with abnormally low glucose concentration and exacerbated by culture with high glucose concentration [7, 8]. This reflects caloric restriction (CR) and caloric excess, respectively. CR has been used as an experimental intervention to delay the onset and consequences of aging, although evidence of its effectiveness in human subjects is limited [9]. A strategy to circumvent poor compliance in keeping to CR regimes *in vivo* is the development of caloric restriction mimetics - small molecules that produce similar effects on ageing and senescence delay as CR without limiting caloric intake. The mechanistic basis of this remains unclear [10].

A long-held concept in theories of cell senescence is the increased wear-and-tear of cell proteins leading to increased formation of reactive oxygen species (ROS) and related increased steady-state levels of oxidative damage to proteins and DNA – with telomere attrition associated with the latter [11, 12]. Development of small-molecule antisenescent compounds has often focussed on activators of transcription factor nuclear factor erythroid 2-related factor 2 (Nrf2). Nrf2 regulates the cellular expression of a battery of protective genes countering oxidative stress, environment toxic insults, and lipid peroxidation [13]. Increased activation of Nrf2 may thereby decrease macromolecular damage and delay senescence. Nrf2 is a constitutive translocational oscillator, sensing challenge to homeostasis in the cell cytoplasm and activating a proportionate protective transcriptional response [14]. Silencing of Nrf2 leads to premature senescence [15]. Activators of Nrf2 increase the expression of genes linked to an antioxidant cytoprotective response, pentose phosphate pathway metabolism, aldehyde metabolism, autophagy, and proteasomal proteolysis and decrease the expression of sterol response element binding protein-1 (SREBF1) and related lipogenic gene expression [13, 14]. No link between Nrf2 and CR in delay of fibroblast senescence has hitherto been made, but herein we reveal that this plays a crucial role.

## 2. Materials and Methods

### 2.1. Cells and Reagents

Human fetal lung MRC-5 fibroblasts and human foreskin BJ fibroblasts were purchased from the European Collection of Animal Cell Cultures (ECACC, Porton Down, London, UK). MRC-5 and BJ cells were grown in Eagle's Minimum Essential Medium (MEM; Invitrogen Paisley, Scotland) supplemented with 10% fetal bovine serum (FBS) (Labtech International Ltd., Uckfield, UK), 1% penicillin-streptomycin (Sigma-Aldrich, Poole, Dorset, UK), 2 mM L-glutamine, and 100 mM sodium pyruvate (Invitrogen). R-Sulforaphane (SFN, ≥95%), L-lactic dehydrogenase (rabbit muscle), pepsin (porcine stomach mucosa), pronase E (*Streptomyces griseus*), leucine aminopeptidase type VI (porcine kidney) and prolidase (from porcine kidney, specific activity 145 units/mg protein), L-lactic acid, senescence cell staining kit (cat. no. CS0030), and glucose assay kit (cat. no. GAHK20) were from Sigma-Aldrich (Poole, UK). A Qiagen RNeasy Mini Spin column was purchased from Qiagen (West Sussex, UK). Reagents for microarray analysis were from Agilent Technologies UK Ltd. (Wokingham, Berkshire).

### 2.2. MRC-5 and BJ Fibroblast Culture

MRC-5 and BJ fibroblasts were passaged every 7 days: seeding density - 4000 cells/cm^2^ for MRC-5 fibroblasts and 3000 cells/cm^2^ for BJ fibroblasts. Cell viability was assessed by the Trypan Blue dye exclusion technique. Cells were cultured under 5% CO_2_ in air, 100% humidity at 37°C with Minimum Essential Medium (MEM) supplemented with 10% fetal bovine serum, 2 mM L-glutamine, 1 mM sodium pyruvate, 100 units/mL penicillin, and 100 *μ*g/mL streptomycin. MRC-5 fibroblasts were treated with and without 1 *μ*M R-sulforaphane (SFN, ≥95%) in DMSO (final DMSO concentration 0.002%), added 24 h after the start of each passage, from passage 2 until senescence at passage 12. BJ fibroblasts were treated similarly from passages 3 to 22.

### 2.3. Custom Quantitative mRNA Array and Microarray Analysis

MRC-5 fibroblasts at passage 4 were seeded in 6-well plates (200,000 cells per well). After 24 h, 1 *μ*M SFN or vehicle control (0.002% DMSO) was added and cultures continued for 4, 8, 12, 24, 36, 48, and 72 h. Thereafter, cells were collected and washed twice with ice-cold phosphate-buffered saline. MRC-5 fibroblasts treated chronically with and without SFN from passage 3 to passage 11 were also analysed. Relative mRNA copy number was determined using 600–800 ng total RNA in a custom 50-gene by the NanoString method [16], outsourced to NanoString (Seattle, USA). References genes were *β*-actin (ACTB), clathrin heavy chain 1 (CLTC), and *β*-glucuronidase (GUSB). Total RNA (600 ng) from the same samples was used for microarray analysis, analysing the expression of 20,773 genes. The hybridization sample was loaded into an Agilent SureHyb chamber/SureHyb gasket slide and heated at 65°C for 17 h at 10 rpm. Slides were washed, dried, and scanned using an Agilent scanner and data extracted using Agilent Feature Extraction Software v11.5. There were 3 biological replicates and one technical replicate. Spot intensities were log_2_-transformed and normalized and differences assessed by Student's *t*-test.

### 2.4. Real-Time Quantitative Reverse Transcription PCR (qRT-PCR)

Reverse transcriptase reaction was performed with 100 ng (BJ cells) or 50 ng (MRC-5 cells) total RNA and using High-Capacity cDNA Reverse Transcription Kit (Applied Biosystems™). The reaction was incubated at 25°C for 10 min, then 37°C for 2 h, and then 85°C for 5 min. After 2-fold dilution, 2 *μ*L reverse transcription product cDNA was used for qRT-PCR by the SYBR Green technique with SYBR® Green JumpStart™ *Taq* ReadyMix (S4438 Sigma) on an ABI 7500 real-time PCR system. The reaction volume was 15 *μ*L, temperature 95°C for 2 min, followed 40 cycles at 95°C for 15 s and 60°C for 1 min. The primers were as follows: TXNIP forward: 5′-CTGGCGTAAGCTTTTCAAGG-3′, reverse: 5′-AGTGCACAAAGGGGAAACAC-3′; HK1 forward: 5′-CTGCTGGTGAAAATCCGTAGTGG-3′, reverse: 5′- GTCCAAGAAGTCAGAGATGCAGG-3′; and HK2 forward 5′- TCCACTCCTCTCAGCATTGG-3′, reverse 5′-CTTGCCCCATTATCCCATGC-3′. The quantification for each gene expression level was evaluated using a standard curve. Gene expression was normalized to ribosomal protein L19 (MRC fibroblasts) or ACTB (BJ fibroblasts).

### 2.5. Western Blotting

The cell protein extract was prepared with RIPA buffer (20 mM Tris-HCl, pH 7.5, 150 mM NaCl, 1 mM, Na_2_EDTA, 1 mM EGTA, 1% NP-40, 1% sodium deoxycholate, 2.5 mM sodium pyrophosphate, 1 mM *β*-glycerophosphate, 1 mM Na_3_VO_4_, and 1 *μ*g/mL leupeptin) containing a protease inhibitor cocktail and phosphatase inhibitor cocktail (Cell Signaling Technology #9806). Cell protein (20 *μ*g) was electrophoresed on 10% polyacrylamide gels, transferred to a polyvinylidene difluoride membrane, and blocked with 3% (*w*/*v*) bovine serum albumin (BSA) in Tris-buffered saline with Tween 20 (TBST): 10 mM Tris/HCl, pH 7.5, 150 mM NaCl, and 0.05% Tween 20. Membranes were blotted with a primary antibody at 4°C overnight, washed with TBST, and a secondary antibody was added for 1 h at room temperature. After washing, immunoreactivity was detected using enhanced chemiluminescence using visualization with GNOME XRQ NPC (Syngene, Cambridge, UK). Protein band intensities were quantified by ImageQuant TL software (GE Healthcare, Little Chalfont, UK). For the reference protein, *β*-actin, membranes were stripped with a stripping buffer (100 mM 2-mercaptoethanol, 2% SDS, and 62.5 mM Tris/HCl, pH 6.8), blocked with 3%(*w*/*v*) BSA in TBST, and re-probed with an anti-*β*-actin antibody.

### 2.6. Other Analysis

Protein glycation, oxidative damage and N*_ε_*(*γ*-glutamyl)lysine (GEEK) adducts of cell protein and related excreted free adducts, 8-oxodG excreted into culture medium, and reduced glutathione (GSH) content of MRC-5 cells were assayed by stable isotopic dilution analysis liquid chromatography-tandem mass spectrometry as described [17–20].

### 2.7. Statistical Analysis

Experiments were performed using ≥3 biological replicates. Data are presented as mean ± SD. Significance testing was performed using Student's *t*-test for 2 data group comparisons and one-way ANOVA for 4 group comparisons; ANOVA repeated measures analysis was used for repeated observations of cells of common lineage and treatment. Bonferroni corrections were applied for transcriptomics microarray and quantitative mRNA custom array (NanoString) data. Median growth inhibitory GC_50_ values of SFN were determined by nonlinear regression of viable cell number (V) data on SFN concentration, fitting to the equation *V* (%of control) = 100 × GC_50_^*n*^/(GC_50_^*n*^ + [SFN]^*n*^), and solving for GC_50_ and logistic regression coefficient, *n*. Statistical significance was performed using the Statistical Package for the Social Sciences (SPSS) Version 24.0 (SPSS Inc., Chicago, USA). Significance was defined as *P* < 0.05.

## 3. Results

### 3.1. Delay of Replicate Senescence in Human MRC-5 and BJ Fibroblasts by Low Concentration Sulforaphane

**Figure 1:**
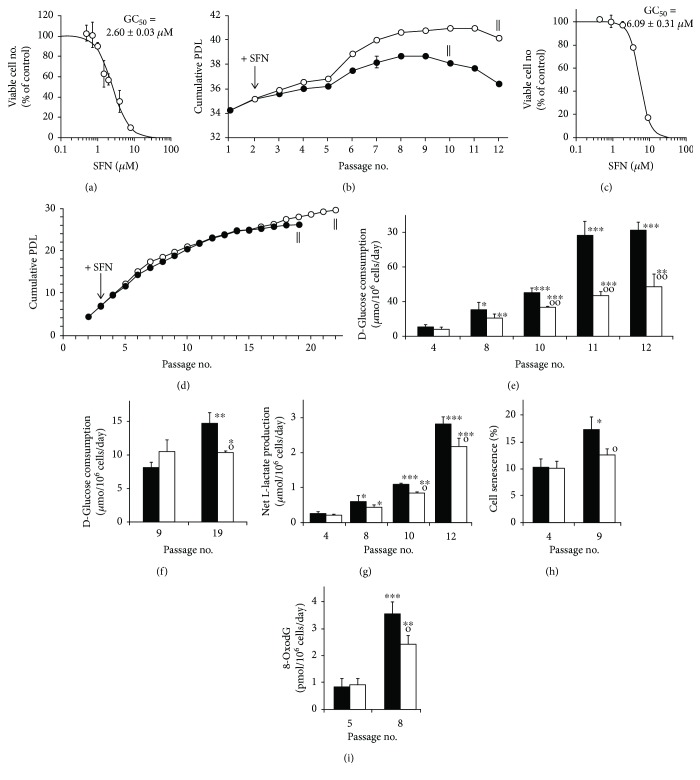
Delay of replicative senescence in MRC-5 and BJ fibroblasts by sulforaphane and link to suppression of glucose consumption. (a) Concentration-response curve for the effect of SFN on the growth of MRC-5 fibroblasts *in vitro*. The median growth inhibitory concentration GC_50_ of SFN was 2.60 ± 0.03 *μ*M and *n* logistic regression coefficient *n* = 2.02 ± 0.03. (b) Growth of MRC-5 fibroblasts to senescence with delay by SFN. ‖ indicates when cells stopped growing and thereafter decreased in viability (ΔPDL < 0). Treatment effect, *P* = 2 × 10^−5^, and time × treatment interaction, *P* = 0.004; *repeated measures*. (c) Concentration-response curve for the effect of SFN on the growth of BJ fibroblasts *in vitro*. GC_50_ of SFN was 6.09 ± 0.31 *μ*M and *n* = 3.04 ± 0.05. (d) Growth of BJ fibroblasts to senescence. Treatment effect (*P* = 0.002) and time × treatment interaction (*P* = 0.009; *repeated measures*). Key: ↓ initiation of treatment with 1 *μ*M SFN (○▬○) and control, +0.002% DMSO (●▬●), one treatment per passage. (e, f) Glucose consumption in selected passages of MRC-5 and BJ fibroblast cultures from experiments in (b) and (d). For MRC-5 cells, treatment effect, *P* = 0.003, and treatment × time interaction, *P* = 0.004; *repeated measures*. For MRC-5 cultures: (g) net production of L-lactate (treatment effect, *P* = 0.003; *repeated measures*); (h) *β*-galactosidase-stain senescence; and (i) flux of formation of DNA oxidation adduct, 8-oxodG, in cell cultures from experiment in (b). Key: solid bars, control; unfilled bars, +1 *μ*M SFN. Significance: ^∗^, ^∗∗^, and ^∗∗∗^, *P* < 0.05, *P* < 0.01, and *P* < 0.001 with respect to cPDL of untreated control at passage 4 control of MRC-5 fibroblasts (e, g–i) and passage 9 control of BJ fibroblasts (f); o and oo, *P* < 0.05 and *P* < 0.01 with respect to untreated control of the same passage; *t*-test; *N* = 3 except *N* = 21 and *N* = 17 for determination of GC_50_ values for SFN in MRC-5 and BJ cells, respectively.

When human MRC-5 fibroblasts were incubated in primary culture with SFN, growth arrest and toxicity was induced at ≥2 *μ*M with a median growth inhibitory GC_50_ concentration of 2.60 ± 0.03 *μ*M (Figure 1(a)). Culture of MRC-5 fibroblasts through 12 passages led to senescence with a maximum cumulative population doubling level (cPDL) of 38.7 ± 0.1 (*n* = 3) at passage 8. Concurrent treatment with 1 *μ*M SFN at the start of each passage from passage 2 delayed maximum cPDL to passage 10 to 40.9 ± 0.1—an increase in cPDL of 2.2 (*n* = 3, *P* < 0.001) with mean ΔcPDL 1.9, with respect to the untreated control (Figure 1(b)). In four independent replicate studies, the increase in cPDL achieved with SFN treatment above the control was 2.0 ± 0.3 (*P* < 0.001). A similar effect was found with human BJ fibroblasts where SFN had a GC_50_ of 6.09 ± 0.31 *μ*M (Figure 1(c)) and treatment with 1 *μ*M SFN at the start of each passage from passage 3 led to delayed and increased maximum cPDL at passage 22 of 29.8 ± 0.1—a maximum cPDL increase of 3.5 (*n* = 3, *P* < 0.001) (Figure 1(d)). A feature of the delay in senescence induced by SFN hitherto not recognised is the suppression of senescence-associated increased glucose consumption. As senescence approaches in MRC-5 fibroblasts, there was a 3-fold increase in glucose consumption. Treatment with SFN progressively suppressed this increase by 19% at passage 4 to 53% at passage 12 (Figure 1(e)). A similar effect was found in the delay of senescence by SFN in BJ fibroblasts, albeit a decrease in glucose consumption occurring at late passages when senescence was delayed (Figure 1(f)). There was also a decrease in net L-lactate formation (Figure 1(g)) and onset of increased cell senescence - as indicated by decreased cell staining for *β*-galactosidase activity with SFN treatment (Figure 1(h)). Also, on the approach to senescence in MRC-5 fibroblasts, the flux of formation of the DNA oxidative damage marker 8-oxodeoxyguanosine (8-OxodG) was increased by ca. 4-fold from passages 5 to 8 which was partly prevented by treatment with SFN (Figure 1(i)). Hence, there is evidence of increased genomic oxidative damage concomitant with the earliest stages of increased glucose metabolism in MRC-5 fibroblasts.

### 3.2. Effect of Sulforaphane on Antioxidant Response Element-Linked and Senescence Associated Gene Expression in Human MRC-5 Fibroblasts during Replicative Senescence

**Figure 2:**
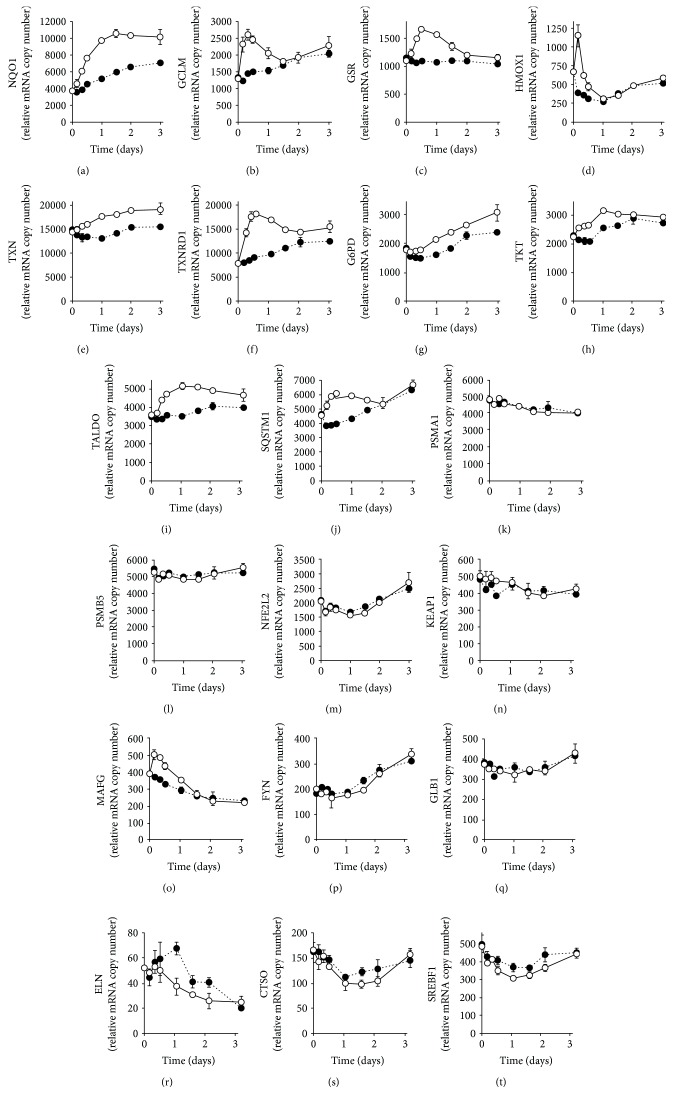
Changes in gene expression induced by sulforaphane in early passage, nonsenescent MRC-5 cells *in vitro*. Key: + 1 *μ*M SFN (○▬○) and control, + 0.002% DMSO (●····●). (a) NQO1 (*P* = 2 × 10^−4^, *P*′ = 0.003, and *P* ^″^ = 0.006), (b) GCLM (*P* = 0.003, *P*′ = 0.020, and *P* ^″^ = 0.005), (c) GSR (*P* = 0.017, *P*′ = 0.006, and *P* ^″^ = 0.019), (d) HMOX1 (*P* = 0.006, *P*′ = 0.011, and *P* ^″^ = 0.014), (e) TXN (*P* = 0.005, *P*′ = 0.008, and *P* ^″^ = 0.034), (f) TXNRD1 (*P* = 8 × 10^−4^, *P*′ = 0.004, and *P* ^″^ = 0.004), (g) G6PD (*P* = 0.002, *P*′ = 0.013), (h) TXT (*P* = 0.003, *P*′ = 0.001, and *P* ^″^ = 0.018), (i) TALDO (*P* = 0.004, *P*′ = 0.004, and *P* ^″^ = 0.034), (j) SQSTM1 (*P* = 7 × 10^−4^, *P*′ = 0.002, and *P* ^″^ = 0.004), (k) PSMA1 (*P* = 0.015), (l) PSMB5, (m) NFE2L2 (*P* = 0.008), (n) KEAP1 (*P* = 0.045), (o) MAFG (*P* = 3 × 10^−4^, *P*′ = 0.011, and *P* ^″^ = 0.031), (p) FYN (*P* = 0.001), (q) GLB1 (*P* = 0.017), (r) ELN (*P* = 0.012), (s) CTSO (*P* = 0.024), and (t) SREBF1 (*P* = 0.014, *P*′ = 0.041). Data are mean ± SD, *N* = 3. Significance: *P*, *P*′, and *P* ^″^, significance values for time, SFN treatment, and time × SFN treatment effects, respectively (*n* = 48); *ANOVA repeated measures*.

SFN is an activator of transcription factor Nrf2 [14] which regulates antioxidant response element- (ARE-) linked gene expression. SFN-induced activation of Nrf2 in early passage, nonsenescent MRC-5 fibroblasts, produces the expected increase in ARE-regulated antioxidant gene expression: NQO1, GLCM, GSR, HMOX1, TXN, and TXNRD1 (Figures 2(a)–2(f)). There was also an induction of ARE-linked gene expression in the pentose phosphate pathway: G6PD, TXT, and TALDO (Figures 2(g)–2(i)). There was an increased expression of ARE-linked SQSTM1, also called p62, which promotes autophagy but no change in the expression of ARE-regulated subunits of the proteasome (Figures 2(j)–2(l)). There was no change in the expression of Nrf2 and proteins implicated in the regulation of Nrf2 activation – KEAP1 and FYN [14] – although there was a SFN-induced increase in the expression of small maf protein MAFG (Figures 2(m)–2(p)). Treatment with SFN did not decrease the expression of senescence marker *β*-galactosidase (GLB1). There was no effect of SFN on the expression of elastin (ELN) and cathepsin O (CTSO), but there was a decrease in the expression of sterol regulatory element-binding protein 1 (SREBF1) which may contribute to the suppression of cell senescence [21] (Figures 2(q)–2(t)). SFN also induced minor increases in ARE-linked genes FTH1, PDRX1, GCLC, MRP2, and AKR1C1 and a minor decrease in NFKB3 (RelA/p65) (Figure S1). p65 expression, as part of the NF-*κ*B system, is a key positive regulator of the senescence-associated secreted phenotype [22].

**Figure 3:**
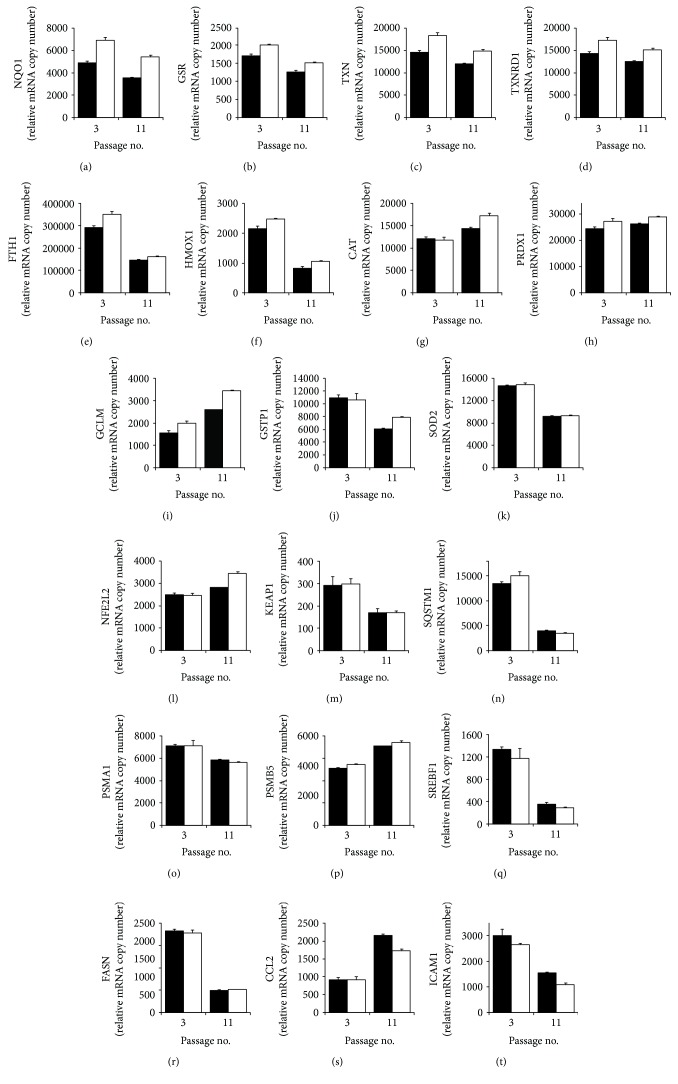
Effect of sulforaphane on gene expression in young and senescent MRC-5 fibroblasts *in vitro*. Key: solid bars, control; unfilled bars, +1 *μ*M SFN. (a) NQO1 (*P* = 0.004, *P*′ = 0.004), (b) GSR (*P* = 2 × 10^−4^, *P*′ = 0.012), (c) TXN (*P* = 0.007, *P*′ = 0.006), (d) TXNRD1 (*P* = 0.031, *P*′ = 0.006), (e) FTH1 (*P* = 0.001, *P*′ = 0.010, and *P* ^″^ = 0.032), (f) HMOX1 (*P* = 0.001, *P*′ = 0.008), (g) CAT (*P* = 0.012, *P*′ = 0.035, and *P* ^″^ = 0.037), (h) PDRX1 (*P*′ = 0.006), (i) GCLM (*P* = 3 × 10^−5^, *P*′ = 0.003), (j) GSTP1 (*P* = 0.003), (k) SOD2 (*P* = 0.001), (l) NFE2L2 (*P* = 0.008, *P*′ = 0.001, and *P* ^″^ = 0.009), (m) KEAP1 (*P* = 0.005), (n) SQSTM1 (*P* = 0.001, *P*′ = 0.009, and *P* ^″^ = 0.007), (o) PSMA1 (*P* = 0.013), (p) PSMB5 (*P* = 2 × 10^−4^, *P*′ = 0.002), (q) SREBF1 (*P* = 0.003), (r) FASN (*P* = 3 × 10^−5^), (s) CCL2 (*P* = 0.001, *P* ^″^ = 0.002), and (t) ICAM-1 (*P* = 0.003, *P*′ = 0.024). Significance: *P*, passage number effect; *P*′, +SFN effect; and *P* ^″^, passage number × SFN effect; *ANOVA repeated measures*.

We next compared the expression of ARE-linked genes and senescent-related genes in nonsenescent and senescent MRC-5 fibroblasts – cells from passages 3 and 11, respectively – and the effect of SFN treatment. Relative mRNA copy number assessment showed decreased expression of redox-related genes, NQO1, GSR, TXN, and TXNRD1, in senescence with increased expression by SFN in both nonsenescent and senescent fibroblasts, correcting the decrease in senescence (Figures 3(a)-3(d)). For FTH1 and HMOX1, expression was decreased in senescence and SFN increased the expression in both nonsenescent and senescent fibroblasts but failed to correct the decrease in senescence completely (Figures 3(e) and 3(f)). For other redox genes, CAT, PDRX1, and GCLM, expression was increased in senescence and treatment with SFN increased the expression further (Figures 3(g)-3(i)). Expression of other redox genes, GSTP1 and SOD2, was decreased in senescence and not increased by SFN (Figures 3(j) and 3(k)). The expression of Nrf2 was increased in senescence and increased further by SFN, whereas the expression of antagonist KEAP1 was decreased in senescence and unchanged by SFN (Figures 3(l) and 3(m)). There was a decreased expression in autophagy marker SQSTM1 and proteasome subunit PSMA1 in senescence which was not corrected by SFN, whereas the expression of proteasome subunit PSMB5 was increased in senescence and increased further by SFN (Figures 3(n)-3(p)). There was a decreased expression in lipogenic gene expression in senescence, SREBF1 and FASN, suggesting that SREBF1 was likely not a driver of MRC-5 fibroblast senescence; these effects were unchanged by SFN (Figures 3(q) and 3(r)). SFN decreased senescence-associated inflammation, as indicated by partial reversal of the senescence-associated increased expression of monocyte chemoattractant protein-1 (MCP-1; gene CCL2) and decrease in intracellular adhesion molecule-1 (ICAM-1) expression (Figures 3(s) and 3(t)).

Considering extracellular matrix-related genes, SFN surprisingly increased the expression of matrix metalloproteinase- (MMP-) 1 and pro-*α*1(I) chain of collagen-1 in senescence. Increased MMP-1 by SFN may inactivate the SASP and thereby contribute to senescence delay. MMP-3 was characteristically increased in senescence whereas expression of MMP-13 was unchanged (Figure S2, a-d). The expression of CDKN1A (p21) was unchanged and SERPINB2 expression was decreased in senescence, suggesting that CDKN1A and SERPINB2 may not contribute to MRC-5 fibroblast senescence although they were implicated in other models of cellular senescence [23]. Expression of NFKB1 and NFKB3 of the NF-*κ*B inflammatory signaling system was unchanged in MRC fibroblast senescence and was also unchanged by SFN treatment (Figure S2, e–h). For aldehyde metabolism, AKR1C1 expression was decreased in senescence and increased by SFN in both nonsenescent and senescent cells. HIF1A was increased by SFN in senescent cells only. Expression of the following genes was unchanged in senescence and by SFN treatment: SOD1, GCLC, GXP1, MRP2, GLO1, MAFG, ELN, and TLR4 (Figure S2, i-r).

### 3.3. Mechanism of Decrease in Senescence-Associated Increased Glucose Consumption in MRC-5 and BJ Fibroblasts by Sulforaphane

**Figure 4:**
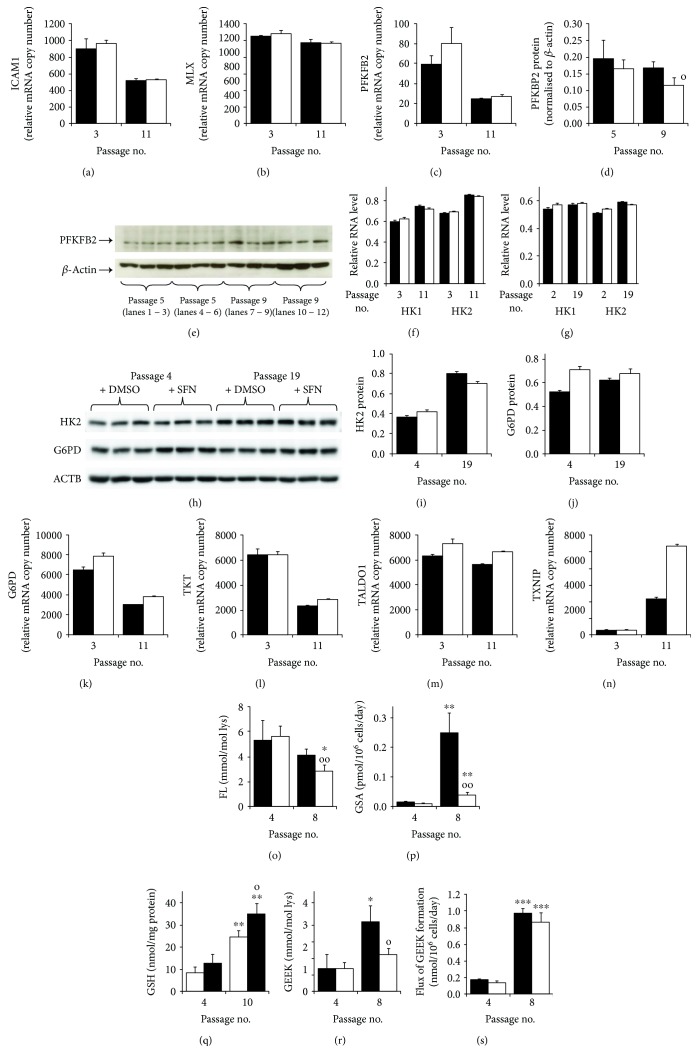
Mechanism of decreased glucose consumption by sulforaphane in delay of fibroblast senescence *in vitro*. Key (bar charts): solid bars, control; unfilled bars, +1 *μ*M SFN. Expression of regulatory glycolysis genes: (a) MLXIP or Mondo A (0.002), (b) MLX (*P* = 0.008), and (c) PFKBP2 (*P* = 0.019) (NanoString analysis). (d, e) PFKBP2 protein – quantitation and related Western blot, respectively. (f) MRC-5 fibroblast HK mRNA: HK1 (*P* = 0.008, *P* ^″^ = 0.021) and HK2 (*P* = 6 × 10^−5^, *P* ^″^ = 0.039). (g) BJ fibroblast HK mRNA: HK1 (*P* = 0.04, *P*′ = 0.016) and HK2 (*P* = 0.003, *P* ^″^ = 0.022) (RT-PCR analysis, relative to *β*-actin). (h) Western blotting of cell protein from BJ fibroblasts at passages 4 and 19 for antigens HK2, G6PD, and reference *β*-actin (ACTB). (i) Quantitation of HK2 protein (*P* = 0.002, *P* ^″^ = 0.015) and (j) quantitation of G6PD protein (*P* = 0.036, *P*′ = 0.012, and *P* ^″^ = 0.018) (Western blotting). (k–n) Relative mRNA copy number in MRC-5 fibroblasts: (k) G6PD (*P* = 0.002, *P*′ = 0.005), (l) TKT (*P* = 0.001), (m) TALDO1 (*P* = 0.016, *P*′ = 0.007), and (n) TXNIP (*P* = 1 × 10^−4^, *P*′ = 9 × 10^−4^, and *P* ^″^ = 2 × 10^−4^), Significance: *P*, passage no effect; *P*′, +SFN effect; and *P* ^″^, passage no × SFN effect. (o–s) Protein damage markers and cellular GSH at passages 4 (nonsenescence) and passage 8 - except passage 10 for GSH (approach to senescence). (o) FL content of cell protein. (p) Flux of formation of protein oxidation adduct, GSA. (q) Cellular concentration of GSH. (r) GEEK cross-link content of cell protein. (s) Flux of formation of GEEK. Significance: *P*, passage number effect; *P*′, +SFN effect; and *P* ^″^, passage number × SFN effect; *ANOVA repeated measures*. ^∗^, ^∗∗^, and ^∗∗∗^, *P* < 0.05, *P* < 0.01, and *P* < 0.001 with respect to passage 4 untreated control; o and oo, *P* < 0.05 and *P* < 0.01 with respect to passage 8 untreated control; *t-test*.

To explore the mechanism of SFN-induced decrease in glucose metabolism in the delay of fibroblast senescence, we studied the expression of genes regulating glycolysis and cellular uptake of glucose. The expression of the master regulator of glycolytic enzyme expression Mondo A (MLXIP) was decreased in MRC-5 fibroblast senescence, and this was unchanged by SFN treatment; the expression of its functional complexing partner, max-like protein X (MLX), was unchanged in senescence and by SFN treatment (Figures 4(a) and 4(b)). The relative mRNA copy number of 6-phosphofructo-2-kinase/fructose-2,6-biphosphatase 2 (PFKFB2), controlling levels of glycolytic regulator fructose-2,6-bisphosphate (F-2,6-P_2_), was decreased in MRC-5 fibroblast senescence and was unchanged by SFN treatment. Treatment with SFN, however, decreased PFKFB2 protein in senescent MRC fibroblasts by 31% (Figures 4(c)-4(e)) which serves to curb glycolysis in SFN-treated fibroblasts and may contribute to the delay of senescence.

A further level at which glucose metabolism is regulated is hexokinase (HK) which catalyzes the formation of glucose-6-phosphate (G6P) from glucose – the initial entry of glucose into glycolysis and pentose phosphate pathway metabolism. HK1 and HK2 isozymes are expressed in fibroblasts. HK1 and HK2 mRNA levels were increased in senescent MRC-5 fibroblasts, and this was not corrected by SFN, although there was a time × treatment effect where SFN decreased mRNA of HK1 and HK2 in senescence (Figure 4(f)). In BJ fibroblasts, SFN treatment increased HK1 mRNA modestly in both early and late passages whereas for HK2 it was decreased in senescence (Figure 4(g)). A similar effect was found in BJ fibroblasts where SFN decreased HK2 protein in senescence (Figures 4(h) and 4(i)). In contrast, SFN increased G6PD protein in BJ fibroblasts in both early and late passages (Figures 4(h) and 4(j)). SFN treatment of senescent fibroblasts may therefore decrease glucose metabolism by decreasing the expression of HK2. The concomitant increase in G6PD protein serves to decrease the cellular concentration of G6P - an essential cofactor for nuclear translocation of Mondo A/Mlx complex; SFN thereby suppresses glycolysis at the transcriptional level. A similar effect of increased G6PD expression induced by SFN in senescence was found for MRC-5 fibroblasts, which was associated with effects on other pentose phosphate pathway genes, TXT and TALDO1 (Figures 4(k)–4(m)).

An additional level at which SFN imposes a CR response was through increased expression of thioredoxin-interacting protein (TXNIP). The most profound effect of senescence on the gene expression of MRC-5 cells was the 8-fold increase in the expression of TXNIP. This was increased further to 21-fold by treatment with SFN (Figure 4(n)). Increased TXNIP expression was also found to be the most responsive gene to SFN treatment in MRC-5 fibroblast senescence by transcriptomics analysis – see below. TXNIP is an ARE-linked gene [24]. TXNIP provides a curb on cellular uptake of glucose by stimulating internalisation of glucose transporters expressed in fibroblasts, GLUT1, GLUT3, and GLUT4 in [25–27]. On the approach to senescence, therefore, MRC-5 fibroblasts seek to curb the cellular uptake of glucose by upregulating TXNIP expression. SFN treatment raises this curb further to a 21-fold increase of TXNIP mRNA and thereby likely succeeds. To gain evidence of this, we quantified the glucose-derived glycation adduct, fructosyl-lysine (FL), residue content of cell protein in MRC-5 fibroblasts – providing a summation of glucose exposure over the cell lifespan. The FL residue content of cell protein in MRC-5 fibroblasts was decreased by 22% during the approach to senescence and 49% by treatment with SFN, compared to nonsenescent controls, suggesting that there is a curb on glucose uptake on approach to senescence which is potentiated by SFN treatment (Figure 4(o)).

### 3.4. Prevention of Senescence-Associated Oxidative Damage and Nonsulfhydryl Protein Cross-linking of Cell Protein by Sulforaphane

There were multiple indications of early-stage protection from oxidative damage by SFN treatment: decreased flux of 8-oxodG DNA (Figure 1(i)) and decreased flux of formation of the protein oxidative damage marker, glutamic semialdehyde (GSA), in MRC-5 fibroblast cultures at the early approach to senescence (Figure 4(p)). Surprisingly, the cellular concentration of thiol antioxidant, GSH, was increased ca. 3-fold in MRC-5 fibroblasts at senescence and increased further by SFN treatment – likely linked to an SFN-induced increased expression of GCLM (Figure 3(i)). This increase may, however, be an attempt of cells to counter the increasing oxidative threat from increased flux of ROS from dysfunctional mitochondria where mitochondrial density is also increased 4-fold [28].

SFN also decreased the cell protein content of transglutaminase-produced cross-link GEEK by ca. 45%: 3.15 ± 0.71 versus 1.74 ± 0.29 mmol/mol lys. This likely reflected increased proteolysis and clearance of GEEK-modified proteins since the flux of formation of GEEK was increased 5-fold on approach to senescence and was not decreased by SFN (Figures 4(r) and 4(s)). GEEK was the major protein cross-link detected in MRC-5 cell protein; *cf.* levels of the major protein glycation cross-link glucosepane (0.079 ± 0.016 mmol/mol lys) and oxidative cross-link dityrosine (0.34 ± 0.07 mmol/mol lys) which were unchanged in SFN-treated cells.

### 3.5. Transcriptomic Analysis of Delay of Replicative Senescence in MRC-5 Fibroblasts

Transcriptomic analysis of nonsenescent and senescent MRC-5 fibroblasts showed change in the expression of 106 of 20,773 genes assessed: 59 increased and 47 decreased (Table S1). Treatment with SFN corrected 38 of these expression changes in senescence (Table 1). Among gene expressions increased in senescence and corrected by SFN were pyruvate dehydrogenase kinase 2 (PDK2), linked to increased glycolysis in senescence [29]; pyruvate dehydrogenase phosphatase 2 (PDP2), associated with increased HK2 and PDK4 driving mitochondrial dysfunction in senescence; protein phosphatase 1 regulatory subunit 3G (PPP1R3G), which stimulates glycogen synthase activity; collagen type IV alpha 3-binding protein (COL4A3BP), linked to control of mitochondrial fission and fusion, dysfunction, and mitophagy; and Tax1-binding protein (TAX1BP1), a ubiquitin-binding adaptor protein involved in the negative regulation of NF-*κ*B.

There were 15 genes with expression that changed between SFN-treated senescent and SFN-treated nonsenescent fibroblasts (Table 2); only two of these changes were found between untreated senescent and nonsenescent fibroblasts – increased ACKR4 and MOB4 expression. The highest change in SFN treatment-linked gene expression was increased TXNIP, supporting the large increased expression found in mRNA quantitation by the NanoString method (see above).

## 4. Discussion

We show herein for the first time that SFN, a health beneficial isothiocyanate formed from a glucosinolate precursor in broccoli, delays fibroblast senescence through a CR mimetic-like response. This was achieved by treatment with 1 *μ*M SFN, once per week – a concentration and dose frequency which was nontoxic to fibroblasts and is also achievable clinically [30].

The response was associated with glucose and glycolytic restriction mediated by a multitier mechanism limiting the cellular availability and metabolism of glucose: increased expression of TXNIP, curbing the entry of glucose into cells; decreased HK2, curbing the entry of glucose into cellular metabolism; and decreased PFKFB2 and increased G6PD, downregulating glycolysis. Benefits accrued were protection from increased protein and DNA oxidative damage and GEEK protein cross-linking, which are otherwise part of increased macromolecular wear-and tear driving senescence.

We observed a profound increase in glucose consumption by MRC-5 on the approach to senescence and countering of this by SFN. A similar response was found in BJ fibroblasts. Increased glucose metabolism was mediated by increased HK2 expression at mRNA and protein levels. Increased HK2 mRNA and total HK activity in senescent human fibroblasts was reported previously [31, 32]. Total HK activity is particularly sensitive to HK2 expression; both HK1 and HK2 operate close to saturation with glucose substrate in situ where *k*_cat_ for HK2 is *ca.* 5 times higher than that of HK1 [33]. SFN decreased MRC-5 fibroblast glucose metabolism by HK through multiple reinforcing mechanisms: (i) increased TXNIP expression which decreases the availability of glucose to HK by curbing the cellular uptake of glucose [25–27]; (ii) increased expression of G6PD, decreasing G6P and downregulating transcriptional induction of HK2 by Mondo A/Mlx/G6P complex [34]; and destabilization of the HK2 protein to proteolytic degradation through a decrease in cellular glucose concentration [35] - indicated by decreased FL residue content of cell protein.

Increased cellular HK2 activity on the approach to senescence with cells replete with glucose leads to increased cellular G6P concentration. G6P is the first metabolite of glycolysis and pentose phosphate pathways of glucose metabolism. It is also an essential cofactor for functional activity of the Mondo A/Mlx complex that increases expression of glycolytic enzymes by complex translocation to the nucleus and binding to promoter carbohydrate response elements of target genes, including expression of TXNIP as an autoregulatory curb on glucose metabolism during periods of excess glucose availability [36]. Increased TXNIP expression was found in untreated MRC-5 fibroblasts in senescence, likely occurring as an indirect response to increased glycolysis stimulated by increased HK2 expression. The curb fails as increased glucose metabolism occurs in senescence. Expression of TXNIP in senescence was enhanced further, however, by treatment with SFN.

A further feature of increased glycolysis mediated by HK2 is the accumulation of G6P and partial displacement of HK2 from mitochondria, impairing mitochondrial oxygen consumption similar to that found under conditions of inhibition of ADP recycling [37]. This increases the mitochondrial membrane potential and ROS formation, contributing to previously observed mitochondrial dysfunction and telomere shortening [38, 39]. A marker of HK2 displacement from mitochondria is increased metabolic channelling of G6P to glycogen synthesis [37]. Increased glycogen deposition is a long-established characteristic of senescent fibroblasts [40]. Indeed, MRC-5 fibroblast senescence was associated with increased expression of PPP1R3G herein – a positive regulator of glycogen synthesis, which was corrected by SFN treatment. SFN induced decrease of glucose metabolism by HK2 likely suppressed dysfunction of mitochondria and increased ROS formation – reflected in decreased oxidative damage to DNA and protein.

**Figure 5:**
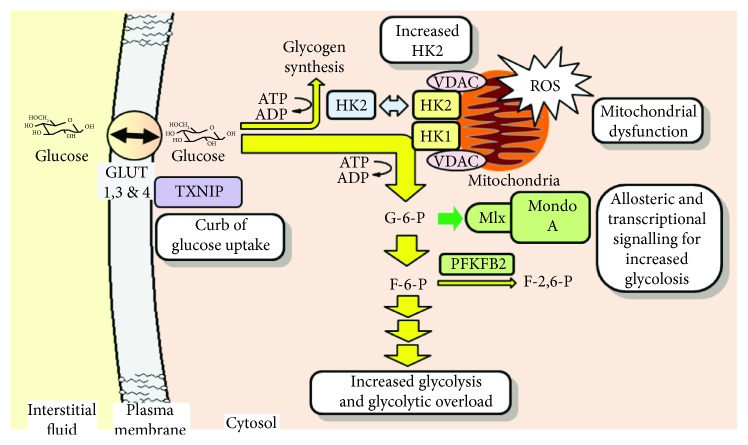
Entry of glucose into glycolysis, hexokinase-2 linked mitochondrial dysfunction, and transcriptional signaling for glycolytic overload. Text boxes indicate processes involved in senescence-associated glycolytic overload. These are reversed by SFN treatment except for TXNIP-mediated curb on glucose uptake which is enhanced. Key: green, transcriptional and allosteric effectors for increased glycolysis; blue, HK2 displacement from mitochondria with increased cellular G6P, with metabolic channelling to glycogen synthesis. VDAC: voltage-dependent anion channel.

Increased glycolysis in fibroblast senescence may be also driven by increased PFKBP2 activity [41], increasing levels of the allosteric positive regulatory cofactor of glycolytic enzymes, F-2,6-P_2_ [42]. SFN treatment decreased PFKBP2 protein in senescent MRC-5 fibroblasts, likely through increased proteasomal proteolysis. This synergises with other glycolysis restriction effects of SFN. The above processes are summarised schematically (Figure 5).

In transcriptomic analysis, SFN treatment corrected 36% of gene expression changes found in senescence. Changes in gene expression related to suppression of increased glycolysis (PDK2, PDP2) improved mitochondrial function (COL4A3BP), increased glycogen synthesis (PPP1R3G), decreased inflammation (TAX1BP1), and increased proteolysis (TRIM63). These are likely linked to the glycolytic restriction and downstream effects of SFN contributing to delay of senescence. The apparent disparity between outcomes in microarray transcriptomics analysis and mRNA copy number array was due to the robust quantitative methodology of the latter technique. Where gene expression changes were large – such as for SFN treatment-associated increase in TXNIP expression in senescent MRC-5 cells – outcomes from the two methods were corroborative.

SFN-induced decrease in cell protein content of the major cellular protein cross-link, GEEK, formed by transglutaminase may also contribute to delay of senescence. Histone proteins are targets for cross-linking in senescence and may thereby impair replicative capacity [43]. Increased cross-linking activity in senescent fibroblasts parallels the increased expression of transglutaminase-2 - the most abundant transglutaminase in fibroblasts with expression increasing 5-fold in senescence as part of the senescence phenotype [44]. This was confirmed as linked to increased protein cross-linking herein by the *ca.* 5-fold increase in flux of formation of GEEK on approach to senescence. SFN corrected the increased cellular protein content of GEEK in senescence by increasing the clearance of GEEK-modified proteins. This was likely mediated by increased cellular proteolysis since flux of GEEK formation remained unchanged. This suggests GEEK cross-linking of cellular proteins may be efficiently removed and excreted by SFN treatment. This cross-linking may contribute to dermal and other fibroblast stiffening with age. For dermal fibroblasts, this is directly linked to altered viscoelastic properties of the collagen matrix and may contribute to the age-related impairment of elastic properties in human skin [45]. The action of SFN in decreasing GEEK protein cross-links may be a route to a dermal rejuvenation effect.

Our experimental findings show evidence of cellular glucose and glycolytic restriction induced by SFN associated with delay of fibroblast senescence. Glucose is a major caloric nutrient source of human fibroblasts in primary culture [46], and therefore, this may also impose CR and SFN thereby achieving a CR mimetic-like response. Whether dietary exposure to SFN *in vivo* imposes a similar CR-like effect is unknown but may now deserve investigation. Glucose and glycolytic restriction of MRC-5 human lung fibroblasts was found to delay senescence previously. This was associated with decreased expression of p16^INK4a^ (p16) – an activator of cell senescence, mediated by chromatin remodeling through effects on histone acetylation and methylation of the p16 promoter where increased expression of sirtuin-1 (SIRT1) was involved [47]. Increased activity of SIRT1 is thought to mediate CR and increased longevity effects of *trans*-resveratrol and similar compounds [48]. This provides a link between cellular glucose and glycolytic restriction effects of SFN found herein and previous approaches for development of CR mimetics [49].

## 5. Conclusions

The results of this study suggest the following conclusions:

1. SFN delays the senescence of human MRC-5 and BJ fibroblasts *in vitro*
2. Cell senescence is associated with a progressive and marked increased rate of glucose metabolism through glycolysis, and this is countered by SFN treatment – glycolytic restriction previously found to delay senescence [7]
3. SFN decreased glucose metabolism on the approach to senescence by increasing the expression of TXNIP, curbing the entry of glucose into cells; decreasing HK2, curbing the entry of glucose into cellular metabolism; decreasing 6-phosphofructo-2-kinase, downregulating the formation of the allosteric enhancer of glycolysis F-2,6-P_2_; and increasing G6PD, downregulating the ChRE-mediated transcriptional enhancement of glycolysis by Mondo/Mlx/G6P
4. SFN also enhanced the clearance of proteins cross-linked by transglutaminase which otherwise increased in senescence
5. Screening of compounds to counter senescence-associated glycolytic overload may be an effective strategy to identify compounds with antisenescence activity

## Figures and Tables

**Table 1:** Gene expression changes in senescence of MRC-5 fibroblasts *in vitro* corrected by treatment with sulforaphane (transcriptomics analysis).

No.	Gene ID	Name	*P* value	ΔLog_2_ expression	Comment
1	NPY	Pro-neuropeptide Y	0.022	6.50	Regulates cell cycle, proliferation.
2	TRIM63	E3 ubiquitin-protein ligase	0.012	5.26	Promotes ubiquitination and degradation of misfolded proteins.
3	BANK1	B-cell scaffold protein with ankyrin repeats	0.003	3.53	Lyn-mediated tyrosine phosphorylation of inositol 1,4,5-trisphosphate receptors
4	LRRC69	Leucine-rich repeat-containing protein 69	0.005	2.97	Unknown function.
5	PPP1R3G	Protein phosphatase 1 regulatory subunit 3G	0.013	2.78	Stimulates glycogen synthase activity.
6	TMEM47	Transmembrane protein 47	0.044	2.69	Senescence associated gene.
7	SLC14A1	Urea transporter 1	0.009	2.57	Facilitates transmembrane urea transport down a concentration gradient.
8	DAP	Death-associated protein 1	0.048	2.49	Negative regulator of autophagy
9	INA	Alpha-internexin	0.031	2.18	Intermediate filament protein.
10	GMFG	Glia maturation factor gamma	0.007	2.08	Target of senescence-associated microRNA, hsa-miR-369-5P.
11	MATN3	Matrilin-3	0.037	1.97	Extracellular matrix protein
12	PSG1	Pregnancy-specific beta-1-glycoprotein 1	0.040	1.96	Senescence-associated protein.
13	DAAM2	Disheveled-associated activator of morphogenesis 2	0.028	1.95	Regulator of the Wnt signaling pathway.
14	PDK2	Pyruvate dehydrogenase kinase, isoenzyme 2	0.038	1.73	Linked to increased glycolysis in senescence.
15	SORT1	Sortilin 1	0.035	1.69	Sorting receptor in the Golgi compartment and clearance receptor on the cell surface.
16	PSG9	Pregnancy-specific beta-1-glycoprotein 9	0.048	1.68	Senescence associated protein.
17	MAP7D3	MAP7 domain-containing protein 3	0.049	1.66	Promotes the assembly and stability of microtubules.
18	GPC4	Glypican-4	0.016	1.60	Cell surface heparan sulfate proteoglycan involved in Wnt signaling.
19	KCTD12	Pfetin	0.016	1.58	Potassium channel protein.
20	UHMK1	Serine/threonine-protein kinase Kist	0.007	1.50	Controls CDKN1B subcellular location and cell cycle progression in the G1 phase.
21	RCAN3	Calcipressin-3	0.039	1.44	Negative regulator of calcineurin-linked transcriptional regulation.
22	PDP2	Mitochondrial pyruvate dehydrogenase-phosphatase 2	0.031	1.34	Drives mitochondrial dysfunction in senescence.
23	MALSU1	Mitochondrial assembly of ribosomal large subunit protein 1	0.049	1.33	Involved in mitochondrial ribosome function and mitochondrial translation.
24	UFD1L	Ubiquitin recognition factor in ER-associated degradation protein 1	0.038	1.25	Upregulated in stress-induced senescence.
25	HLA-E	HLA class I histocompatibility antigen, *α*-chain E	0.002	1.25	Senescent fibroblast marker.
26	NDST2	Bifunctional heparan sulfate N-deacetylase/N-sulfotransferase 2	0.016	1.23	Involved in biosynthesis of heparan sulfate.
27	ZMAT2	Zinc finger matrin-type protein 2	0.001	1.09	Regulated by sirtuin-1.
28	MUC1	Mucin-1	0.015	1.06	Transmembrane glycoprotein participating in growth factor receptor signaling.
29	CTBS	Di-N-acetylchitobiase (EC 3.2.1.-)	0.037	1.04	Involved in the degradation of asparagine-linked glycoproteins
30	ABTB2	Ankyrin repeat and BTB/POZ domain-containing protein 2	0.002	0.99	Substrate adaptor for cullin-3 ubiquitin ligase; may increase Nrf2 degradation in senescence.
31	LYNX1	Ly-6/neurotoxin-like protein 1	0.024	0.85	Negative regulator of nicotinic receptor signaling.
32	DNAJC19	Mitochondrial import inner membrane translocase subunit TIM14	0.008	0.82	Mitochondrial co-chaperone.
33	COL4A3BP	Collagen type IV alpha-3-binding protein	0.001	0.81	Controls mitochondrial fission, fusion, dysfunction, and mitophagy.
34	TMEM165	Transmembrane protein 165	0.014	0.80	Target of miR-181a in fibroblast senescence.
35	TAX1BP1	Tax1-binding protein 1	0.011	0.76	Ubiquitin-binding adaptor protein; negative regulation of the NF-*κ*B.
36	HSD11B1L	Hydroxysteroid 11-beta-dehydrogenase 1-like protein	0.004	0.75	Involved in glucocorticoid metabolism.
37	TMX2	Thioredoxin-related transmembrane protein 2	0.045	0.66	Disulfide isomerase enriched on the mitochondria-associated membrane of the endoplasmic reticulum.
38	ZNF525	Zinc finger protein 525	0.045	0.65	May be involved in transcriptional regulation.

Genes listed had expression changes between passages 3 and 11 in MRC-5 fibroblasts which were corrected by SFN treatment. Gene expression changes are rank-ordered by log_2_ expression change (highest to lowest). *P* values are with Bonferroni-correction applied.

**Table 2:** Gene expression changed in senescence of MRC-5 fibroblasts treated with SFN *in vitro* (transcriptomics analysis).

No.	Gene ID	Name	*P* value	ΔLog_2_ expression
1	TXNIP	Thioredoxin-interacting protein	0.039	6.8
2	ACKR4	Atypical chemokine receptor 4	0.024	5.6
3	C11orf87	Neuronal integral membrane protein 1	0.039	3.8
4	PRRX2	Paired related homeobox 2	0.046	3.3
5	FZD4	Frizzled class receptor 4	0.005	2.5
6	ACAA2	Mitochondrial 3-oxoacyl-coenzyme A thiolase	0.024	2.5
7	MOB4	Mps one binder kinase activator-like 3	0.007	1.8
8	SLC46A1	Proton-coupled folate transporter	0.035	1.7
9	CSNK1G1	Casein kinase 1 gamma 1	0.023	1.5
10	ACTR2	Actin-related protein 2 homolog	0.014	1.1
11	MAEA	Macrophage erythroblast attacher	0.016	1.1
12	GALC	Galactosylceramidase	0.024	1.1
13	VAT1	Vesicle amine transport 1	0.019	1.1
14	ARSJ	Arylsulfatase family member J	0.004	0.9
15	SUN1	Sad1 and UNC84 domain containing 1	0.045	−0.7

Genes listed had expression changes between passages 3 and 11 in MRC-5 fibroblasts treated with SFN. Gene expression changes are rank-ordered by log_2_ expression change (highest to lowest). *P* values are with Bonferroni-correction applied.

## Data Availability

The data used to support the findings of this study are available from the corresponding author upon request.

## Supplementary Materials

Supplementary Materials The supplementary materials include one table and 2 figures. Supplementary Table 1: transcriptomic analysis of genes with changed expression in senescence of MRC-5 fibroblasts in vitro. Supplementary Figure 1: changes in gene expression induced by sulforaphane in early passage, nonsenescent MRC-5 fibroblasts in vitro. Supplementary Figure 2: effect of sulforaphane on end-of-passage gene expression in young and senescent MRC-5 fibroblasts in vitro.

## References

[1] Hayflick L. (1965). The limited *in vitro* lifetime of human diploid cell strains. *Experimental Cell Research*.

[2] Salama R., Sadaie M., Hoare M., Narita M. (2014). Cellular senescence and its effector programs. *Genes & Development*.

[3] Ball A. J., Levine F. (2005). Telomere-independent cellular senescence in human fetal cardiomyocytes. *Aging Cell*.

[4] Xie Z., Jay K. A., Smith D. L. (2015). Early telomerase inactivation accelerates aging independently of telomere length. *Cell*.

[5] Dimri G. P., Lee X., Basile G. (1995). A biomarker that identifies senescent human cells in culture and in aging skin in vivo. *Proceedings of the National Academy of Sciences of the United States of America*.

[6] Childs B. G., Durik M., Baker D. J., van Deursen J. M. (2015). Cellular senescence in aging and age-related disease: from mechanisms to therapy. *Nature Medicine*.

[7] Jin J., Zhang T. (2013). Effects of glucose restriction on replicative senescence of human diploid fibroblasts IMR-90. *Cellular Physiology and Biochemistry*.

[8] Blazer S., Khankin E., Segev Y. (2002). High glucose-induced replicative senescence: point of no return and effect of telomerase. *Biochemical and Biophysical Research Communications*.

[9] Masoro E. J., Yu B. P., Bertrand H. A. (1982). Action of food restriction in delaying the aging process. *Proceedings of the National Academy of Sciences of the United States of America*.

[10] Weindruch R., Keenan K. P., Carney J. M. (2001). Caloric restriction mimetics: metabolic interventions. *The Journals of Gerontology Series A: Biological Sciences and Medical Sciences*.

[11] Sitte N., Merker K., von Zglinicki T., Grune T., Davies K. J. A. (2000). Protein oxidation and degradation during cellular senescence of human BJ fibroblasts: part I--effects of proliferative senescence. *The FASEB Journal*.

[12] Chen Q., Fischer A., Reagan J. D., Yan L. J., Ames B. N. (1995). Oxidative DNA damage and senescence of human diploid fibroblast cells. *Proceedings of the National Academy of Sciences of the United States of America*.

[13] Malhotra D., Portales-Casamar E., Singh A. (2010). Global mapping of binding sites for Nrf2 identifies novel targets in cell survival response through ChIP-Seq profiling and network analysis. *Nucleic Acids Research*.

[14] Xue M., Momiji H., Rabbani N. (2015). Frequency modulated translocational oscillations of Nrf2 mediate the antioxidant response element cytoprotective transcriptional response. *Antioxidants & Redox Signaling*.

[15] Kapeta S., Chondrogianni N., Gonos E. S. (2010). Nuclear erythroid factor 2-mediated proteasome activation delays senescence in human fibroblasts. *The Journal of Biological Chemistry*.

[16] Geiss G. K., Bumgarner R. E., Birditt B. (2008). Direct multiplexed measurement of gene expression with color-coded probe pairs. *Nature Biotechnology*.

[17] Rabbani N., Shaheen F., Anwar A., Masania J., Thornalley P. J. (2014). Assay of methylglyoxal-derived protein and nucleotide AGEs. *Biochemical Society Transactions*.

[18] Thornalley P. J., Waris S., Fleming T. (2010). Imidazopurinones are markers of physiological genomic damage linked to DNA instability and glyoxalase 1-associated tumour multidrug resistance. *Nucleic Acids Research*.

[19] Schäfer C., Schott M., Brandl F., Neidhart S., Carle R. (2005). Identification and quantification of *ε*-(*γ*-glutamyl)lysine in digests of enzymatically cross-linked leguminous proteins by high-performance liquid chromatography−electrospray ionization mass spectrometry (HPLC-ESI-MS). *Journal of Agricultural and Food Chemistry*.

[20] Xue M., Weickert M. O., Qureshi S. (2016). Improved glycemic control and vascular function in overweight and obese subjects by glyoxalase 1 inducer formulation. *Diabetes*.

[21] Kim Y. M., Shin H. T., Seo Y. H. (2010). Sterol regulatory element-binding protein (SREBP)-1-mediated lipogenesis is involved in cell senescence. *Journal of Biological Chemistry*.

[22] Chien Y., Scuoppo C., Wang X. (2011). Control of the senescence-associated secretory phenotype by NF-*κ*B promotes senescence and enhances chemosensitivity. *Genes & Development*.

[23] Hsieh H.-H., Chen Y. C., Jhan J. R., Lin J. J. (2017). The Serine protease inhibitor SerpinB2 binds and stabilizes p21 in senescent cells. *Journal of Cell Science*.

[24] He X., Ma Q. (2012). Redox regulation by nuclear factor erythroid 2-related factor 2: gatekeeping for the basal and diabetes-induced expression of thioredoxin-interacting protein. *Molecular Pharmacology*.

[25] Waldhart A. N., Dykstra H., Peck A. S. (2017). Phosphorylation of TXNIP by AKT mediates acute influx of glucose in response to insulin. *Cell Reports*.

[26] Wu N., Zheng B., Shaywitz A. (2013). AMPK-dependent degradation of TXNIP upon energy stress leads to enhanced glucose uptake via GLUT1. *Molecular Cell*.

[27] Longo N., Bell G. I., Shuster R. C., Griffin L. D., Langley S. D., Elsas L. J. (1990). Human fibroblasts express the insulin-responsive glucose transporter (GLUT4). *Transactions of the Association of American Physicians*.

[28] Passos J. F., Saretzki G., Ahmed S. (2007). Mitochondrial dysfunction accounts for the stochastic heterogeneity in telomere-dependent senescence. *PLoS Biology*.

[29] James E. L., Michalek R. D., Pitiyage G. N. (2015). Senescent human fibroblasts show increased glycolysis and redox homeostasis with extracellular metabolomes that overlap with those of irreparable DNA damage, aging, and disease. *Journal of Proteome Research*.

[30] Clarke J. D., Hsu A., Riedl K. (2011). Bioavailability and inter-conversion of sulforaphane and erucin in human subjects consuming broccoli sprouts or broccoli supplement in a cross-over study design. *Pharmacological Research*.

[31] Yoon I. K., Kim H. K., Kim Y. K. (2004). Exploration of replicative senescence-associated genes in human dermal fibroblasts by cDNA microarray technology. *Experimental Gerontology*.

[32] Zwerschke W., Mazurek S., Stöckl P., Hütter E., Eigenbrodt E., Jansen-Dürr P. (2003). Metabolic analysis of senescent human fibroblasts reveals a role for AMP in cellular senescence. *Biochemical Journal*.

[33] Traut T. (2008). Hexokinase. *Allosteric Regulatory Enzymes*.

[34] Dentin R., Tomas-Cobos L., Foufelle F. (2012). Glucose 6-phosphate, rather than xylulose 5-phosphate, is required for the activation of ChREBP in response to glucose in the liver. *Journal of Hepatology*.

[35] Xia H.-g., Najafov A., Geng J. (2015). Degradation of HK2 by chaperone-mediated autophagy promotes metabolic catastrophe and cell death. *Journal of Cell Biology*.

[36] Stoltzman C. A., Peterson C. W., Breen K. T., Muoio D. M., Billin A. N., Ayer D. E. (2008). Glucose sensing by MondoA:Mlx complexes: a role for hexokinases and direct regulation of thioredoxin-interacting protein expression. *Proceedings of the National Academy of Sciences*.

[37] John S., Weiss J. N., Ribalet B. (2011). Subcellular localization of hexokinases I and II directs the metabolic fate of glucose. *PLoS One*.

[38] Viticchiè G., Agostini M., Lena A. M. (2015). p63 supports aerobic respiration through hexokinase II. *Proceedings of the National Academy of Sciences*.

[39] Passos J. F., von Zglinicki T. (2005). Mitochondria, telomeres and cell senescence. *Experimental Gerontology*.

[40] Robbins E., Levine E. M., Eagle H. (1970). Morphologic changes accompanying senescence of cultured human diploid cells. *The Journal of Experimental Medicine*.

[41] Meacci E., Vannini F., Vasta V., Farnararo M., Bruni P. (1993). Effect of aging on insulin regulation of fructose 2,6-bisphosphate metabolism in human fibroblasts. *Biochemistry and Molecular Biology International*.

[42] Arden C., Tudhope S. J., Petrie J. L. (2012). Fructose 2,6-bisphosphate is essential for glucose-regulated gene transcription of glucose-6-phosphatase and other ChREBP target genes in hepatocytes. *Biochemical Journal*.

[43] Kim J. H., Choy H. E., Nam K. H., Park S. C. (2001). Transglutaminase-mediated crosslinking of specific core histone subunits and cellular senescence. *Annals of the New York Academy of Sciences*.

[44] Park S. C., Yeo E. J., Han J. A. (1999). Aging process is accompanied by increase of transglutaminase C. *The Journals of Gerontology Series A: Biological Sciences and Medical Sciences*.

[45] Schulze C., Wetzel F., Kueper T. (2012). Stiffening of human skin fibroblasts with age. *Clinics in Plastic Surgery*.

[46] Goldstein S., Ballantyne S. R., Robson A. L., Moerman E. J. (1982). Energy metabolism in cultured human fibroblasts during aging in vitro. *Journal of Cellular Physiology*.

[47] Li Y., Tollefsbol T. O. (2011). p16INK4a suppression by glucose restriction contributes to human cellular lifespan extension through SIRT1-mediated epigenetic and genetic mechanisms. *PLoS One*.

[48] Howitz K. T., Bitterman K. J., Cohen H. Y. (2003). Small molecule activators of sirtuins extend Saccharomyces cerevisiae lifespan. *Nature*.

[49] Testa G., Biasi F., Poli G., Chiarpotto E. (2014). Calorie restriction and dietary restriction mimetics: a strategy for improving healthy aging and longevity. *Current Pharmaceutical Design*.

