# Supplementary material for: Sulforaphane Delays Fibroblast Senescence by Curbing Cellular Glucose Uptake, Increased Glycolysis, and Oxidative Damage
Source: Oxid Med Cell Longev. 2018 Nov 22;2018:5642148. doi: 10.1155/2018/5642148 (PMC6282131; doi:10.1155/2018/5642148)
Supplement: Supplementary Materials — The supplementary materials include one table and 2 figures. Supplementary Table 1: transcriptomic analysis of genes with changed expression in senescence of MRC-5 fibroblasts in vitro. Supplementary Figure 1: changes in gene expression induced by sulforaphane in early passage, nonsenescent MRC-5 fibroblasts in vitro. Supplementary Figure 2: effect of sulforaphane on end-of-passage gene expression in young and senescent MRC-5 fibroblasts in vitro. [file 5642148.f1.pdf]

Supplementary figures and tables

Sulforaphane delays fibroblast senescence by curbing cellular glucose uptake, increased glycolysis and oxidative damage

Florence Hariton,[1†] Mingzhan Xue,[1†] Naila Rabbani[1], Mark Fowler[2] and Paul J. Thornalley[1]

TABLE S1: Transcriptomic analysis of genes with changed expression in senescence of MRC-5 fibroblasts *in vitro*.

No	Gene ID	Name	P-value	ΔLog$_2$ expression
1.	NR5A2	Nuclear receptor subfamily 5 group A member 2	0.043	6.83
2.	NPY	Pro-neuropeptide Y	0.022	6.50
3.	DNAJC6	Putative tyrosine-protein phosphatase auxilin	0.002	6.41
4.	APBB1IP	Amyloid beta A4 precursor protein-binding family B member 1-interacting protein	0.007	5.91
5.	ATCAY	Caytaxin	0.019	5.91
6.	ACKR4	Atypical chemokine receptor 4	0.003	5.64
7.	TRIM63	E3 ubiquitin-protein ligase	0.012	5.26
8.	PDE11A	Dual 3',5'-cyclic-AMP and -GMP phosphodiesterase 11A	0.044	5.18
9.	FOXL2	Forkhead box protein L2	0.029	5.01
10.	HSD17B6	17-beta-hydroxysteroid dehydrogenase type 6	0.044	4.68
11.	LYPD5	Ly6/PLAUR domain-containing protein 5	0.020	4.43
12.	TMEM176A	Transmembrane protein 176A	0.020	4.39
13.	C11orf70	Uncharacterized protein C11orf70	0.000	3.87
14.	MMP3	Matrix metalloprpteinase-3	0.010	3.77
15.	SELENOP	Selenoprotein P	0.037	3.63
16.	BANK1	B-cell scaffold protein with ankyrin repeats	0.003	3.53
17.	RGS16	Regulator of G-protein signaling 16	0.011	3.23
18.	CPPED1	Serine/threonine-protein phosphatase CPPED1	0.003	3.10
19.	LRRC69	Leucine-rich repeat-containing protein 69	0.005	2.97
20.	SYPL2	Synaptophysin-like protein 2	0.034	2.92
21.	PPP1R3G	Protein phosphatase 1 regulatory subunit 3G	0.013	2.78
22.	TMEM47	Transmembrane protein 47	0.044	2.69
23.	SLC14A1	Urea transporter 1	0.009	2.57
24.	DAP	Death-associated protein 1	0.048	2.49
25.	STYK1	Aspartyl aminopeptidase	0.017	2.39
26.	D4S234E	Tyrosine-protein kinase STYK1	0.022	2.33
27.	INA	Alpha-internexin	0.031	2.18
28.	GMFG	Glia maturation factor gamma	0.007	2.08
29.	MATN3	Matrilin-3	0.037	1.97
30.	PSG1	Pregnancy-specific beta-1-glycoprotein 1	0.040	1.96
31.	SHC4	SHC-transforming protein 4	0.040	1.95
32.	DAAM2	Disheveled-associated activator of morphogenesis 2	0.028	1.95
33.	MOB4	MOB-like protein phocein	0.000	1.81
34.	PDK2	Pyruvate dehydrogenase kinase, isoenzyme 2	0.038	1.73
35.	SORT1	Sortilin 1	0.035	1.69
36.	PSG9	Pregnancy-specific beta-1-glycoprotein 9	0.048	1.68

TABLE S1: Transcriptomic analysis of genes with changed expression in senescence of MRC-5 fibroblasts *in vitro* (cont'd).

No	Gene ID	Name	P-value	ΔLog_2 expression
37.	MAP7D3	MAP7 domain-containing protein 3	0.049	1.66
38.	GPC4	Glypican-4	0.016	1.60
39.	KCTD12	Pfetin	0.016	1.58
40.	UHMK1	Serine/threonine-protein kinase Kist	0.007	1.50
41.	MANBAL	Protein MANBAL	0.016	1.45
42.	RCAN3	Calcipressin-3	0.039	1.44
43.	PDP2	Mitochondrial pyruvate dehydrogenase-phosphatase 2	0.031	1.34
44.	MALSU1	Mitochondrial assembly of ribosomal large subunit protein 1	0.049	1.33
45.	UFD1L	Ubiquitin recognition factor in ER-associated degradation protein 1	0.038	1.25
46.	HLA-E	HLA class I histocompatibility antigen, α-chain E	0.002	1.25
47.	NDST2	Bifunctional heparan sulfate N-deacetylase/N-sulfotransferase 2	0.016	1.23
48.	ZMAT2	Zinc finger matrin-type protein 2	0.001	1.09
49.	MUC1	Mucin-1	0.015	1.06
50.	CTBS	Di-N-acetylchitobiase (EC 3.2.1.-)	0.037	1.04
51.	ABTB2	Ankyrin repeat and BTB/POZ domain-containing protein 2	0.002	0.99
52.	LYNX1	Ly-6/neurotoxin-like protein 1	0.024	0.85
53.	DNAJC19	Mitochondrial import inner membrane translocase subunit TIM14	0.008	0.82
54.	COL4A3BP	Collagen type IV alpha-3-binding protein	0.001	0.81
55.	TMEM165	Transmembrane protein 165	0.014	0.80
56.	TAX1BP1	Tax1-binding protein 1	0.011	0.76
57.	HSD11B1L	Hydroxysteroid 11-beta-dehydrogenase 1-like protein	0.004	0.75
58.	TMX2	Thioredoxin-related transmembrane protein 2	0.045	0.66
59.	ZNF525	Zinc finger protein 525	0.045	0.65
60.	RAI1	Retinoic acid-induced protein 1	0.048	-0.29
61.	ACAD10	Acyl-CoA dehydrogenase family member 10	0.031	-0.64
62.	MTRF1	Peptide chain release factor 1, mitochondrial	0.009	-1.24
63.	OXR1	Oxidation resistance protein 1	0.009	-1.47
64.	COLEC11	Collectin-11	0.003	-1.78
65.	ABCC3	Canalicular multispecific organic anion transporter 2	0.019	-1.80
66.	PDE4DIP	Myomegalin	0.001	-1.86
67.	MT-ND1	Mitochondrially encoded NADH:Ubiquinone Oxidoreductase Core Subunit 1	0.022	-1.86
68.	PRPF39	Pre-mRNA-processing factor 39 (PRP39 homolog)	0.021	-1.88
69.	TRIM45	Tripartite motif-containing protein	0.014	-1.94
70.	ZNF169	Zinc finger protein 169	0.035	-1.95

TABLE S1: Transcriptomic analysis of genes with changed expression in senescence of MRC-5 fibroblasts *in vitro* (cont'd).

No	Gene ID	Name	P-value	ΔLog$_2$ expression
71.	LAT	Linker for activation of T-cells family member 1	0.037	-1.95
72.	PPM1N	Probable protein phosphatase 1N (EC 3.1.3.16)	0.010	-2.09
73.	AMPD3	AMP deaminase 3	0.017	-2.10
74.	NKTR	NK-tumor recognition protein	0.024	-2.33
75.	HIST2H2AC	Histone H2A type 2-C	0.006	-2.36
76.	COL23A1	Collagen alpha-1(XXIII) chain	0.014	-2.41
77.	GOLGA6L9	Golgin subfamily A member 6-like protein 9	0.028	-2.41
78.	L2HGDH	L-2-hydroxyglutarate dehydrogenase, mitochondrial	0.042	-2.44
79.	NUTM2G	NUT family member 2G	0.047	-2.47
80.	TBC1D3G	TBC1 domain family member 3G	0.031	-2.86
81.	ABCC10	Multidrug resistance-associated protein 7	0.026	-2.89
82.	ADHFE1	Hydroxyacid-oxoacid transhydrogenase, mitochondrial	0.022	-2.92
83.	BCOR	BCL-6 corepressor	0.014	-2.95
84.	FBXL19-AS1	Long non-coding RNA FBXL19-AS1	0.035	-2.96
85.	SCARNA13	Small Cajal Body-Specific RNA 13	0.002	-2.98
86.	CXCL2	C-X-C motif chemokine 2	0.000	-3.04
87.	SLC25A16	Solute carrier family 25 member 16	0.038	-3.11
88.	HIST1H4A	Histone H4	0.022	-3.16
89.	SNORD114-26	Small Nucleolar RNA, C/D Box 11426	0.016	-3.23
90.	FBXL17	F-box/LRR-repeat protein 17	0.021	-3.32
91.	SAP25	Histone deacetylase complex subunit SAP25	0.036	-3.41
92.	CTSL	Cathepsin L1	0.044	-3.46
93.	SNORD114-17	Small Nucleolar RNA, C/D Box 114-17	0.035	-3.51
94.	SNORD114-3	Small Nucleolar RNA, C/D Box 1143	0.009	-3.54
95.	MALAT1	Long non-coding RNA 47	0.036	-3.78
96.	FOSB	Protein fosB (G0/G1 switch regulatory protein 3)	0.016	-3.92
97.	CCDC144A	Coiled-coil domain-containing protein 144A	0.003	-3.93
98.	SMA4	β-Glucuronidase-like protein SMA4	0.033	-4.06
99.	CASKIN2	Caskin-2 (CASK-interacting protein 2)	0.030	-4.39
100.	RPL23AP32	Ribosomal Protein L23a Pseudogene 32	0.043	-4.43
101.	ANKRD36	Ankyrin repeat domain-containing protein 36A	0.046	-4.62
102.	CISH	Cytokine-inducible SH2-containing protein	0.047	-5.36
103.	MIAT	Long non-coding RNA 66	0.050	-5.71
104.	RNU6ATAC	U6atac Small Nuclear RNA	0.038	-5.82
105.	SNORD12C	Small Nucleolar RNA, C/D Box 12C	0.007	-6.02
106.	NAMPT	Nicotinamide phosphoribosyltransferase (Visfatin)	0.028	-6.27

TABLE S1: Transcriptomic analysis of genes with changed expression in senescence of MRC-5 fibroblasts *in vitro* (cont'd).

Genes listed had expression changes between passages 3 and 11 in MRC-5 fibroblasts. Gene expression changes are rank ordered by $\log_2$ expression change (highest to lowest). P-values are with Bonferroni-correction applied.

Legends to supplementary figures

FIGURE S1: Changes in gene expression induced by sulforaphane in early passage, non-senescent MRC-5 cells *in vitro*. Key: + 1 µM SFN (○▬○) and control, + 0.002% DMSO (●····●). (a) CAT (P = 0.002), (b) SOD1 (P = 0.028), (c) SOD2 (P = 1 x 10^{-4}), (d) FTH1 (P = 0.005, P' = 0.021), (e) GPX1 (P = 5 x 10^{-5}, P" = 0.024), (f) PRDX1 (P' = 0.028), (g) GCLC (P = 0.008, P' = 0.023), (h) GSTP1 (P = 0.006), (i) MRP2 (P' = 0.010), (j) FASN (P = 0.002), (k) CCL2 (P = 0.013), (l) (P = 5 x 10^{-4}), (m) IL1B, (n) IL15, (o) TLR4, (p) NFKB1, (q) NFKB3 (P = 0.003, P' = 0.015), (r) MMP3 (P = 2 x 10^{-4}), (s) MMP13, (t) SERPINE1 (P = 0.002), (u) AKR1C1 (P' = 0.013), (v) CDKN1A, (w) (P = 1 x 10^{-5}), (x) (P = 0.033), (y) GLO1 (P = 0.002), (z) PFKFP4 (P = 0.003), (aa) SERPINB2 (P = 2 x 10^{-4}), (ab) ACTB (P = 0.002, P' = 5 x 10^{-4}, P" = 0.042), (ac) CLTC (P = 0.004) and (ad) GUSB (P = 0.012). Data are mean ± SD, N = 3. Significance: P, P' and P", significance values for time, SFN treatment and time x SFN treatment effects, respectively.

FIGURE S2: Effect of sulforaphane on end-of-passage gene expression in young and senescent MRC-5 fibroblasts *in vitro*. Key: Solid bars, control; unfilled bars + 1 µM SFN. (a) MMP1 (P = 0.002, P' = 0.002, P" = 2 x 10^{-4}), (b) COL1A1 (P" = 0.011), (c) MMP3 (P = 0.001), (d) MMP13, (e) CDKN1A, (f) SERPINB2 (P = 0.002), (g) AKR1C1 (P = 2 x 10^{-4}, P' = 0.002, P" = 0.003), (h) HIF1A (P"= 2 x 10^{-4}), (i) SOD1(P = 0.011), (j) GCLC, (k) GPX1 (P = 0.023), (l) MRP2/UGT1A1 (P = 0.017), (m) GLO1, (n) MAFG (P = 0.007), (o) ELN (P = 0.014), (p) TLR4 (P = 0.031), (q) NFKB1 (P = 0.009, P" = 0.041), (r) NFKB3, (s) GLB1 (P = 0.023) and (t) CDKN1A (P = 0.022, P" = 0.012). Significance: P, passage no effect; P', +SFN effect; and P", passage no x SFN treatment effect.

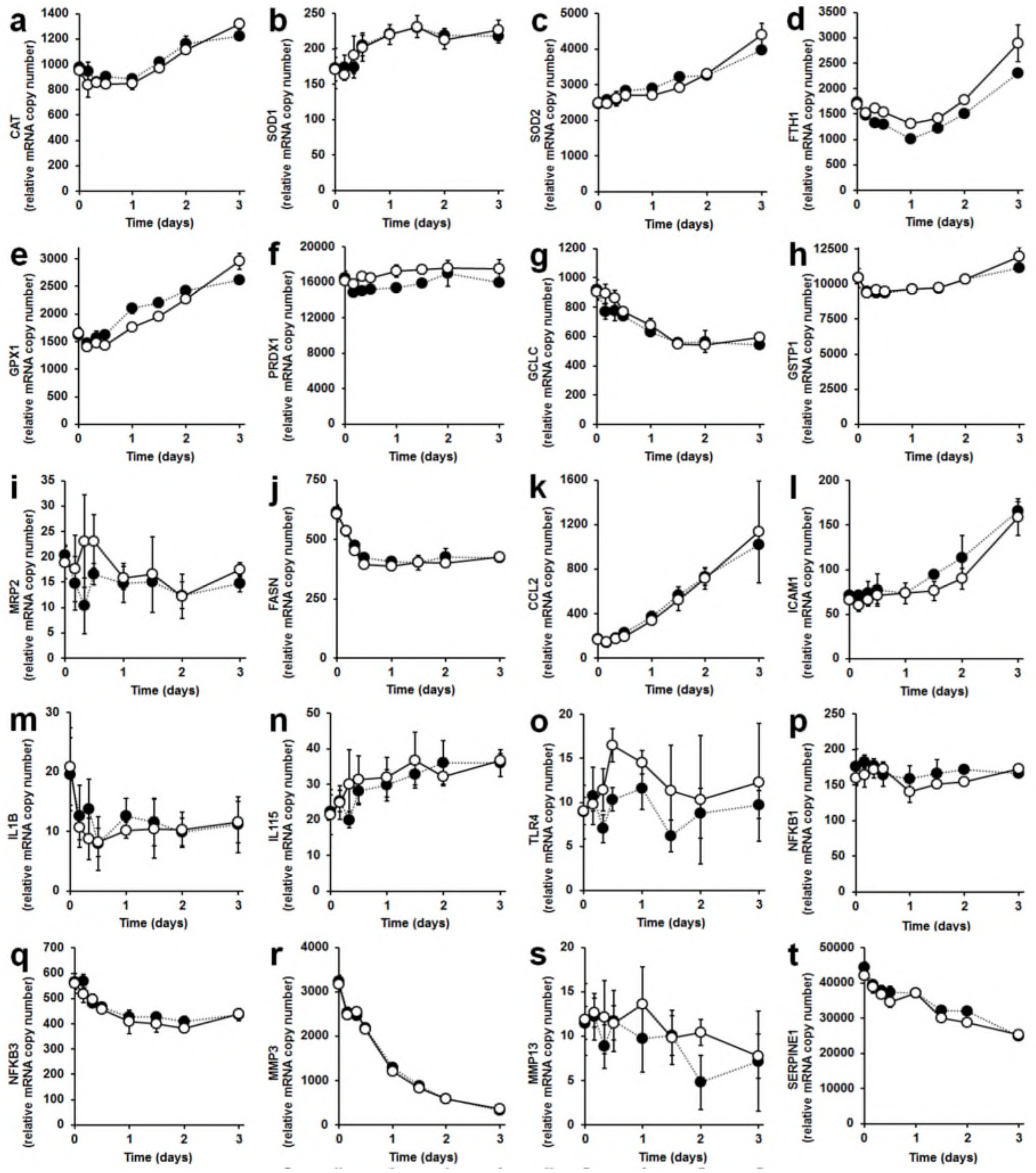
a
CAT
b
SOD1
c
SOD2
d
FTH1
e
GPX1
f
PRDX1
g
GCLC
h
GSTP1
i
MRP2
j
FASN
k
CCL2
l
ICAM1
m
IL1B
n
IL115
o
TLR4
p
NFKB1
q
NFKB3
r
MMP3
s
MMP13
t
SERPINE1
(relative mRNA copy number)
Time (days)

Figure S1

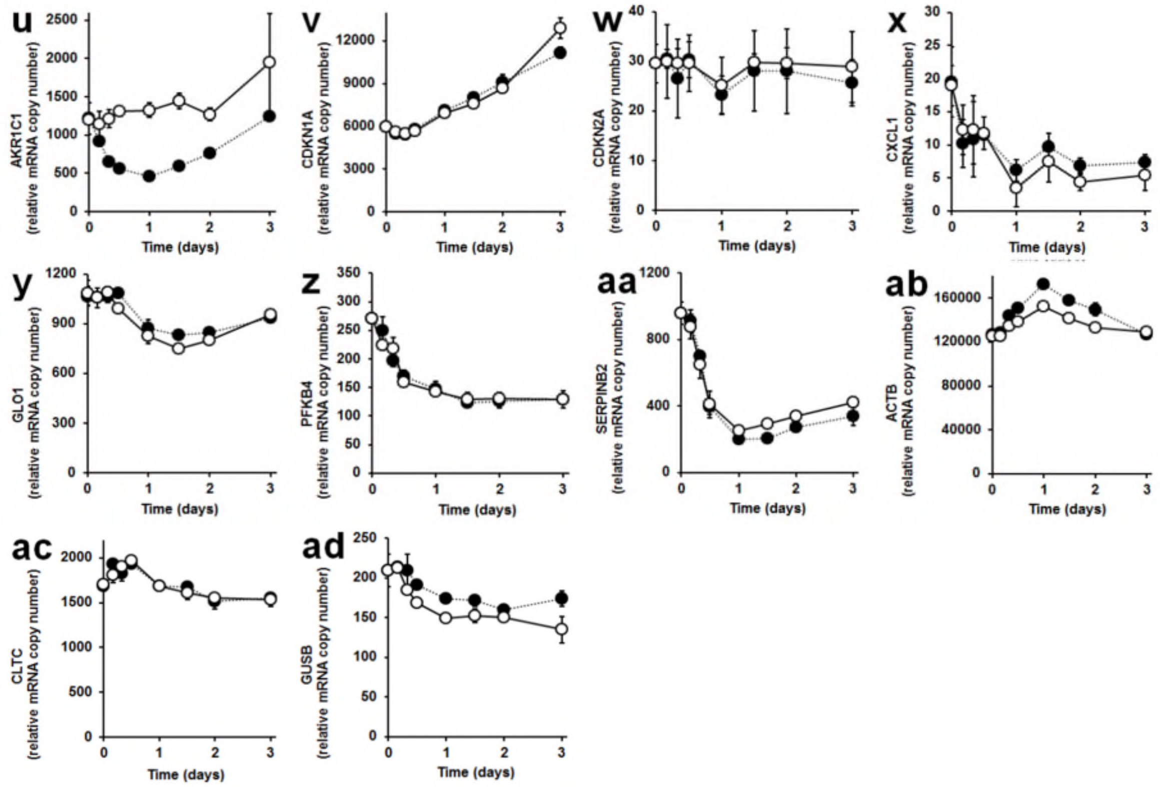

Figure S1 (cont’d)

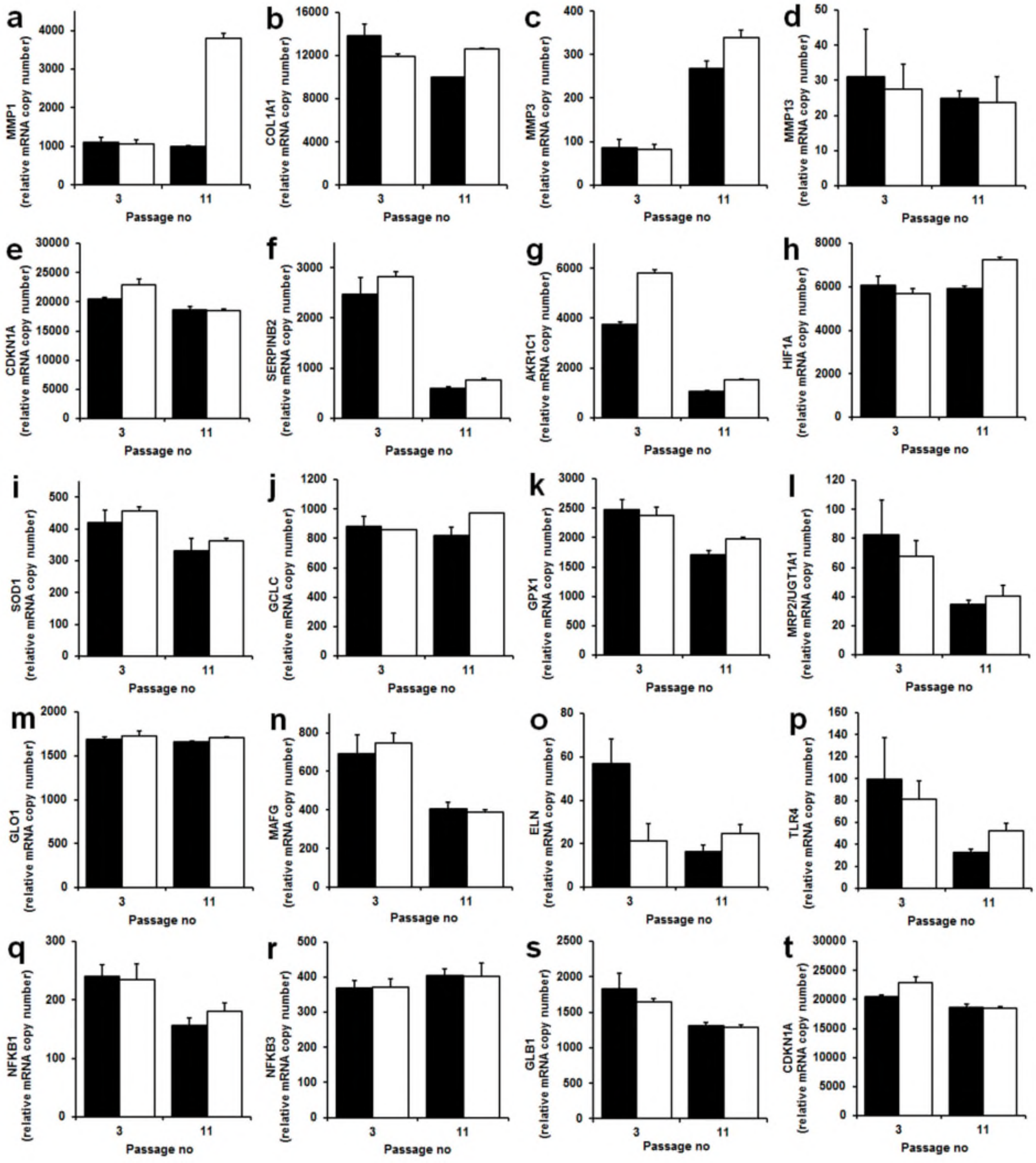
a
MMP1
(relative mRNA copy number)
0
1000
2000
3000
4000
3
11
Passage no
b
COL1A1
(relative mRNA copy number)
0
4000
8000
12000
16000
3
11
Passage no
c
MMP3
(relative mRNA copy number)
0
100
200
300
400
3
11
Passage no
d
MMP13
(relative mRNA copy number)
0
10
20
30
40
50
3
11
Passage no
e
CDKN1A
(relative mRNA copy number)
0
5000
10000
15000
20000
25000
30000
3
11
Passage no
f
SERPINB2
(relative mRNA copy number)
0
1000
2000
3000
3
11
Passage no
g
AKR1C1
(relative mRNA copy number)
0
2000
4000
6000
3
11
Passage no
h
HIF1A
(relative mRNA copy number)
0
2000
4000
6000
8000
3
11
Passage no
i
SOD1
(relative mRNA copy number)
0
100
200
300
400
500
3
11
Passage no
j
GCLC
(relative mRNA copy number)
0
200
400
600
800
1000
1200
3
11
Passage no
k
GPX1
(relative mRNA copy number)
0
500
1000
1500
2000
2500
3000
3
11
Passage no
l
MRP2/UGT1A1
(relative mRNA copy number)
0
20
40
60
80
100
120
3
11
Passage no
m
GLO1
(relative mRNA copy number)
0
500
1000
1500
2000
3
11
Passage no
n
MAFG
(relative mRNA copy number)
0
200
400
600
800
3
11
Passage no
o
ELN
(relative mRNA copy number)
0
20
40
60
80
3
11
Passage no
p
TLR4
(relative mRNA copy number)
0
20
40
60
80
100
120
140
160
3
11
Passage no
q
NFKB1
(relative mRNA copy number)
0
100
200
300
3
11
Passage no
r
NFKB3
(relative mRNA copy number)
0
100
200
300
400
500
3
11
Passage no
s
GLB1
(relative mRNA copy number)
0
500
1000
1500
2000
2500
3
11
Passage no
t
CDKN1A
(relative mRNA copy number)
0
5000
10000
15000
20000
25000
30000
3
11
Passage no

Figure S2
